# The Need for Monetary Financing of Corona Budget Deficits

**DOI:** 10.1007/s10272-020-0885-1

**Published:** 2020-06-07

**Authors:** Paul De Grauwe

**Affiliations:** European Institute, London School of Economics and Political Science, Houghton Street, WC2A 2AE London, UK

## Abstract

Sooner or later, the ECB must accept that monetary financing in support of deficit spending is a necessity not just for mitigating the coronavirus crisis, but also for averting a downward deflationary cycle that could pull the eurozone apart.

The coronavirus pandemic has triggered a combined negative supply and demand shock of unprecedented intensity. Both are having a significant impact on the production of goods and services, and because everyone's income ultimately derives from production, household incomes are quickly falling. With many economies already in a downward spiral and heading towards recession, the danger is that the downturn will become a self-perpetuating and ever-deepening rout.

The twin supply and demand shocks are likely to trigger many 'domino effects'. Companies with large fixed costs that suffer a sudden fall in income are facing financial difficulties, or even bankruptcy. When that happens, the banks and other entities that have lent money to these companies will also be in trouble. That is why massive economic shocks can often lead to banking crises.

But the dominoes do not stop falling there. Governments, too, can face fiscal dangers when they step in to mitigate the crisis. In the case of the current pandemic, national governments will need to save businesses from bankruptcy by granting financial support and subsidies, assist workers by funding temporary unemployment schemes and possibly even come to the rescue of large banks. Worse, all of this must be done at a time of declining tax revenues, which means that government deficits and public debt levels will skyrocket.

We saw how these domino effects work during the 2007-08 financial crisis. The difference now is that the initial shock did not start in financial markets and then spill over into the real economy. Rather, today's shocks emerged from the real economy and are toppling financial markets. But, as in the past, this crisis demands urgent measures to put more space between the falling dominoes. Think of it as macroeconomic 'social distancing'.

## Crisis demands government interventions that increase budget deficits and public debt

What would this look like in practice? First, national governments must intervene on a massive scale to provide financial support for distressed companies and households whose earnings are at risk. Most European governments already seem to be willing to do this. The problem is that large-scale fiscal expansions by eurozone member states could prove tricky, even if the Commission has tabled the activation of the general escape clause of the Stability and Growth Pact (SGP). It is thus critical that the European Central Bank (ECB) steps in to prevent the last domino – member state governments – from falling.

Because they have no choice but to support failing companies, illiquid banks and struggling households, national governments could be entering dangerous territory. The more their debt increases, the greater the risk that their bondholders will panic, as we saw during the 2010-12 sovereign debt crisis. And the countries experiencing the largest debt increase as a result of the coronavirus crisis – Italy, Spain, and France – are three of the four largest eurozone economies.

## ECB should provide monetary financing of corona-induced budget deficits

To head off the risk of a bond market panic, the ECB announced its readiness to buy up distressed governments' bonds. During the 2012 crisis, the ECB laid the groundwork for such a response with its outright monetary transactions program (OMT). The ECB went a step further in April 2020 by dropping all conditionality attached to the use of OMT. This was the correct decision. Yet it is insufficient. The ECB must go further, by preparing to buy government bonds in *primary* markets, effectively issuing money to finance member states' budget deficits during the crisis.

The virtue of such a monetary financing is that it spares national governments from having to issue new debt. Because all new debt would be monetised, the crisis would not increase government debt-to-GDP ratios. For those countries suffering the worst of the pandemic, the threat of a bondholder panic will be removed from the equation. In addition, when the epidemic disappears it will not a leave permanent legacy of unsustainable levels of government debt.

The monetary financing of corona-induced budget deficits is a form of 'helicopter money', i.e. the central bank provides cash to firms and households using the government budget as an intermediary. This is also the appropriate way to organise the distribution of helicopter money. It relies on the government to decide which households and which firms will receive the cash. The government is the appropriate institution as it is vested with the democratic legitimacy to organise such a distribution of cash. This distribution method  is certainly superior to the many proposals that are being made today whereby each citizen would receive the same amount of cash (€1,000, for example). Such a cash distribution would be very ineffective to counter the deflationary spiral because most of the cash would go to households that have not seen their revenues decline. They would likely hoard the largest part of the cash handout. Conversely those who are in greatest need, e.g. the temporarily unemployed, and who are now forced to reduce their consumption would not receive a sufficient amount of cash. A uniform cash handout would be hugely expensive in budgetary terms and mostly ineffective in stopping the deflationary dynamics.

## Objections to monetary financing

There are many objections one could raise to this proposal of monetary financing. As a legal matter, the Treaty on the Functioning of the European Union forbids the ECB from engaging in monetary financing of national budget deficits (i.e. subscribing to newly issued bonds in the primary markets). I trust that EU lawyers, with their unbounded ingenuity, could surely find a way around this restriction. One way this could be done is by national governments issuing perpetual bonds with a zero interest rate that would be presented in the primary market and bought by financial institutions. The latter would sell them to the ECB after a short delay in the secondary markets. There are probably better ways to circumvent the prohibition of monetary financing. The important thing is to realise that in existential crisis situations, governments should use all instruments available to avert catastrophes. A self-imposed rule of no-monetary financing must then be set aside. Or as Cicero put it: *Salus populi, suprema lex* (the welfare of the people is the supreme law).

One might also object on the grounds that monetary financing would produce inflation. Yet under the current circumstances, the inflationary risks are non-existent. If anything, Europe is now facing a deflationary spiral; monetary financing of budget deficits is the only way to stop this spiral. As soon as the deflationary dynamic had been stopped, the ECB will surely halt its monetary financing. In fact, one can expect that in this respect, the ECB is a more credible institution than most national central banks. It is certainly politically more independent than, say, the Bank of England, which can be forced by the UK government to provide monetary support. The ECB, in contrast, cannot be forced by any member state government to continue to provide monetary financing to national governments. Thus, it appears that in the eurozone there is a stronger guarantee that the central bank will quickly want to return to a stability-oriented monetary policy.

A third objection is that monetary financing is not really that different from government debt. When the central bank buys government bonds, it substitutes these bonds for monetary liabilities. The latter consist mainly of reserves that banks hold at the central bank. These reserves are remunerated with an interest rate by the central bank. Thus one type of interest bearing liability issued by the government is substituted for another type of interest bearing liability issued by the central bank. As a result, in both cases, the liabilities of the consolidated public sector will require a debt service (including interest payments). This may not be much of a problem today when the interest rates are close to zero. But it will become one in the future when the central bank wants to fight inflation by raising the interest rate.

What to think of this objection? The problem with it is that it pretends that the remuneration of bank reserves is a necessary feature of central banking. It is not. Until recently, central banks did not pay interest on bank reserves, much in the same way as they do not pay interest on banknotes. The remuneration of bank reserves is undesirable and should be abolished. When the central bank creates money, it generates 'seigniorage', i.e. a profit that arises from the fact that the government has granted a monopoly right to the central bank to create money (money base). This seigniorage should, therefore, be transferred in its entirety to the Treasury and thus to the taxpayer. There is no good reason why part of this monopoly profit should be distributed to commercial banks. Central banks should return to the historical practice of not remunerating bank reserves. In such a regime, there is a big difference between government bonds and money base. The former is an interest bearing liability of the public sector; the latter is not.

## Time to think outside the box

Sooner or later, the ECB must accept that monetary financing in support of deficit spending is a necessity not just for mitigating the coronavirus crisis, but also for averting a downward deflationary cycle that could pull the eurozone apart. It is time to think outside the box and to set aside dogmas that may be appropriate in normal times but not when we face an existential crisis. When the existence of a market system is at stake, all instruments that are available and that can be used to avert disasters should be on the table.
